# Triadic male-infant-male interaction serves in bond maintenance in male Assamese macaques

**DOI:** 10.1371/journal.pone.0183981

**Published:** 2017-10-18

**Authors:** Josefine Kalbitz, Oliver Schülke, Julia Ostner

**Affiliations:** 1 Department of Behavioral Ecology, Johann-Friedrich-Blumenbach Institute for Zoology and Anthropology, University of Göttingen, Göttingen, Germany; 2 German Primate Centre, Leibniz Institute of Primate Research, Göttingen, Germany; 3 Department of Cognitive Ethology, German Primate Centre, Leibniz Institute of Primate Research, Göttingen, Germany; Tierarztliche Hochschule Hannover, GERMANY

## Abstract

While the ultimate consequences of social bonds start to be better understood, the proximate behavioural mechanisms underlying the formation and maintenance of these close affiliative relationships have received less attention. We investigated the possible function of male-infant-male interactions (MIMIs) in male-male social bonding processes by analysing about 9000h of focal animal observations collected on two groups of wild Assamese macaques. In support of an agonistic buffering function of MIMIs, after engaging in a MIMI upon approach, subordinates stayed longer in close proximity of a dominant male. Overall, the frequency of MIMIs increased the stronger the affiliative relationship between two males, suggesting that MIMIs like grooming function in relationship maintenance. We did not find support for a role of MIMIs in bond formation as the frequency of MIMIs did not affect the time a male dyad spent in proximity in the consecutive year. Our results contribute to the general debate on behaviours influencing social dynamics in group living mammals.

## Introduction

The benefits associated with the formation of close affiliative relationships in gregarious species range from selective tolerance for access to resources [1–4], to higher social status via coalition formation [5–8], to cooperative hunting [9, 10], protection against harassment [11], food sharing and mating access [12, 13]. Since many of these benefits concern resource acquisition, social tolerance and agonistic support, close affiliative relationships can buffer individuals against social and environmental stressors [14, 15] whereas the ultimate function is to increase reproductive success and longevity [7, 16–19]. Thus, the development of close affiliative relationships with specific group members is thought to be an adaptive strategy in both sexes and among the sexes and independent of dispersal patterns [13, 20, 21].

Affiliative relationships have been described to vary in terms of value, compatibility and security [12, 13] or similarly in strength, equitability and stability (e.g. [17, 22]). Strong variations in relationship strength between group members have been reported in many primate species (e.g. bonnet macaques, *Macaca radiata* [23]; yellow baboons, *Papio cynocephalus*, [24]; bonobos, *Pan paniscus*, [25]; female black howler monkeys, *Alouatta pigra*, [26]). The equitability of affiliative relationships is often associated with relationship strength (e.g. female chacma baboons, *P*. *ursinus*, [27]; gray-cheeked mangabeys, *Lophocebus albigena*, [28]; female yellow baboons, [29]; vervet monkeys, *Chlorocebus pygerthrus*, [11]; male chimpanzees, *P*. *troglodytes*, [30]), and a few studies have shown that some of those relationships can last over many years (e.g. female yellow baboons, [29]; male chimpanzees, [22, 31]; Assamese macaques, *M*. *assamensis*, [32, 33]). Affiliative relationships which are strong, stable, and equitable are labelled as social bonds [21].

The evolution of differentiated male affiliative relationships in primates is puzzling since male relationships are generally competitive, aggressive, and intolerant pertaining to their competition over a non-sharable resource, fertile females [34–38]. Yet, in the past twenty years a few studies on primate male-male relationships in species with male dispersal, revealed that males differentiate among group members and engage in reciprocal affiliative interactions and thus form social bonds (e.g. Barbary macaques, *M*. *sylvanus*, [39]; Assamese macaques [33]; bonnet macaques [40]; Costa Rican squirrel monkeys, *Saimiri oerstedi*, [41]; chimpanzees [22]).

The structure, stability, and benefits of social bonds are starting to be better understood, whereas the behavioural, physiological and cognitive mechanisms underlying the formation and maintenance of social bonds have received less attention. Close spatial proximity is the basic precondition to engage in exchanges of affiliative behaviours and is regarded as an important component of relationship quality measures. Given the strict hierarchical dominance structure in most mammal groups (e.g. wolves, *Canis lupus*, [42]; non-human primates [43–46]; elephants, *Loxodonta africana*, [47]), lower ranking individuals often face the risk of being aggressed when approaching higher ranking individuals. Therefore, several behavioural patterns including facial expressions, vocalizations, body postures and gestures have evolved to appease the social counterpart in the prelude to an affiliative interaction (e.g. [48–54]).

Appeasement can also be achieved by using infants as a social tool to reduce the risk of aggression from higher ranking males as observed in several cercopithecine species (macaques: [55–58]; *Papio spp*: [59, 60]; geladas, *Theropithecus gelada*, [61]). In male-infant-male interactions (hereafter MIMIs), the interaction between two males is mediated by the infant. After one male carried an infant to a second male or a male with an infant has been approached by a second male both males focus their attention and actions on the infant by lifting the infant, teeth-chattering at it, uttering appeasing vocalisations like grunts and girneys [49, 62], pulling on arms and legs, or inspecting its genitals during which males may make body contact themselves that may last beyond the duration of the MIMI [56, 63, 64]. The context and the exact form of MIMIs seem to differ between species [65]. In baboons [59, 60] and geladas [61] infants are typically handled by males during agonistic encounters, whereas MIMIs [66] in macaques, also called as bridging behaviour [67, 64] or triadic infant interaction [68], occur mainly in affiliative contexts (e.g. Barbary macaques [56, 66, 69]; Stumptail macaques, *M*. *arctoides*, [67]; Tibetan macaques, *M*. *tibetana*, [68]).

MIMIs may function as agonistic buffers between two adult males, i.e. they may reduce the likelihood of subordinates to receive aggression from a higher ranking male when in close proximity [56, 69, 70–72]. Accordingly, MIMIs can increase the chance of being in close proximity with a higher ranking male and with it the likelihood of engaging in affiliative contact [64]. Regulating proximity via MIMIs may, therefore, not only buffer males against aggression, but may also shape male-male affiliative relationships via initiating and/or maintaining social bonds [65, 73, 74]. To better understand the proximate mechanisms involved in bond formation and maintenance, we studied MIMIs in wild adult male Assamese macaques, a species in which some dyads of males form strong, long lasting affiliative relationships [33] which might enable males to engage in risky rank-changing coalitions to rise in rank and thus have better access to fertile females [7].

In our study, we first examined whether the occurrence of a MIMI had a positive effect on the time a subordinate spent in close proximity to a dominant male after an approach and how this effect may be modulated by the strength of the affiliative relationship between the males involved. We predicted that if MIMIs function to maintain close affiliative relationships, the frequency of MIMIs should increase with the strength of the males’ affiliative relationship. To rule out that the correlation between MIMIs and relationship strength results from males engaging randomly in MIMIs with males that are spatially close, we controlled our analysis for the time the dyad spent within 5m spatial proximity. Finally, we predicted, that, if MIMIs function to establish close affiliative relationships, the frequency of MIMIs should be positively correlated to the time two males will spend in close proximity in the future.

Thus, we addressed questions related to the agonistic buffering function of MIMIs [64] while also considering them as a potential bonding mechanism [65]. We assumed that the strength of affiliative relationships is negatively associated with the rate of aggression exchanged between partners [3, 75–78]. Therefore, we predicted that if MIMIs only function as an agonistic buffer, time spent in close proximity after an approach should be more strongly affected by the occurrence of a MIMI the weaker the affiliative relationships between the males. In contrast, if MIMIs play a role in relationship formation and/or maintenance, we predicted that this behaviour increases the time two males spend in proximity irrespective of their relationship strength.

## Methods

### Study site and subjects

The study was carried out on two multi-male, multi-female groups of Assamese macaques living in a hilly, dense and mostly dry evergreen forest which is subject to a shorter dry and an intense monsoon season [79]. The study site is located at Huai Mai Sot Yai in the Phu Khieo Wildlife Sanctuary (PKWS; 16°5′–35′N, 101°20′–55′E) which is part of the contiguous and well-protected ca. 6500 km^2^ Western Isaan forest complex in north-eastern Thailand [79]. Data were collected almost daily from October 2006 until September 2013 of all adult males of the group AS. On average±SD this group consisted of 51.4±4.7 group members, 13±1.9 adult females and 10.1±1.9 adult males. From May 2012 until September 2013 data were collected also from the group AO. This group consisted on average±SD 45.1±2.0 members, 10.6±0.5 adult females and 10.6±0.5 adult males. The group composition varied due to immigration, emigration and death. Throughout the entire study period 17 different adult males lived in the group AS and 10 different adult males in the group AO. All individuals of both groups were well habituated and individually known by all human observers.

### Data collection

Using 20 and later 30min focal animal sampling, behavioural data were collected on adult male macaques of both groups, yielding a total of 8,952 focal observation hours (AS: 7,200h; AO: 1,752h). We recorded continuously for the focal animal the identity of interaction partners and approaches into and departures from close proximity (<1.5m) [80] as well as all affiliative (grooming, body contact, triadic infant handling), submissive (bare teeth, give ground, make room) and aggressive (lunge, slap, chase, push and pull) behaviours with details on duration for time spent in proximity, body contact and grooming (see [46]). Agonistic interactions between males other than the focal animal were recorded ad libitum [81]. Instantaneous scan sampling was used to record all individuals within 5m of the focal individual every 10min [80].

### Data analysis

For the purpose of this study, we calculated dyadic relationship strength with the Composite Sociality Index (CSI [24]) for one year blocks and defined each block as one observation period from the beginning of the mating season (October) until the end of the following non-mating season (September) [82]. The first period of data on group AO spanned over a 5 month period only. The CSI was calculated from six affiliative behaviours, the duration of close spatial proximity [DP], body contact [DB] and grooming [DG] as well as the frequency of close proximity [FP], body contact [FB] and grooming [FG]) using the formula:

$$CSI=\left[\frac{\frac{{FP}_{ij}/h}{{FP}_{ave}/h}+\frac{{FB}_{ij}/h}{{FB}_{ave}/h}+\frac{{FG}_{ij}/h}{{FG}_{ave}/h}+\frac{{DP}_{ij}/h}{{DP}_{ave}/h}+\frac{{DB}_{ij}/h}{{DB}_{ave}/h}+\frac{{DG}_{ij}/h}{{DG}_{ave}/h}}{6}\right]$$

For each dyad and behaviour the duration or frequency per hour was divided by the group mean of this rate for this behaviour. Afterwards the values of all behaviours were summed up and divided by the number of behaviours used (here six behaviours) [33]. By definition the CSI group mean across all dyads in one period is 1, the minimum is zero and the stronger the relationship the higher the CSI [24]. Using Spearman rank correlations for a row-wise matrix correlation, we found all components to be highly correlated in pair-wise comparisons (mean rho_rw,ave_ = 0.92±0.01; range rho_rw,ave_ = 0.88±0.97) [33].

We determined the dominance hierarchy across males for each observation period from decided dyadic agonistic interactions. We used a winner/loser matrix of these interactions (for more details see [33]) to calculate the standardized normalized David’s Score (nDS, in DomCalc [83]). We used the differences in David’s Scores between two males as a measure of rank distances.

Following Ogawa [64], to test the effect of MIMIs on subsequent time spent in close proximity, we used a subset of the data where only subordinate males approached males higher in rank. We counted all observed MIMIs which occurred within the first 3 minutes after a subordinate male approached or until one male of the dyad departed before the 3rd minute. All approaches occurring within the last 3 minutes of the focal protocol were excluded. Descriptive statistic of proximity times and MIMI occurrence are given in the result section.

To assess the impact of MIMIs on the immediate proximity time between two males after a subordinate male approached, we used a Linear Mixed Model (LMM, Model 1) with Gaussian error structure. Time spent in close proximity (≤1.5m) was set as the response and MIMI as a categorical predictor (two levels, MIMI occurred or not). Time spent in MIMI was always short (2-3sec) and was therefore not subtracted from the time spent in proximity which averaged 120±2.78sec. In order to investigate whether the effect of MIMI on proximity time is modulated by the strength of the males’ affiliative relationship, we added the interaction between CSI and MIMI occurrence in the model. Rank distance and ID of the social group were included as control fixed factor and the identities of the initiator and receiver, the dyads as well as the study periods were included as random factors. To control for variation in observation time for each dyad we incorporated dyadic observation time as an offset term. Dyadic observation time was log transformed to fulfil the assumption of the LMM on symmetric distribution of the factors.

We ran the same LMM (Model 2) to assess the impact of MIMIs on the immediate proximity time between two males after a dominant male approached a subordinate male.

It was not possible to assess the effect of MIMIs on the occurrence of aggression after approaches because aggression was so rare that models never stabilized.

To investigate whether the CSI affected the frequency of MIMIs we used a generalized linear mixed model (GLMM; Model 3) with a Poisson dristribiution. The number of MIMIs was set as the response, dyadic CSI values as predictor and the ID of the social group as a control fixed factor. We included proximity (time the focal individual spent within 5m distance with the other) as an offset term to create a rate of MIMI per dyad and the time they spent in 5m proximity. We incorporated dyads, male identities and the observation periods as random factors to control for non-independence of repeated measures across the same individuals within the same periods and the same social groups. In addition, we used observation level random effects to account for overdispersion in count data [84]. Both predictors were power-transformed with 0.25 to achieve a normal distribution.

Finally, to test the long-term effect of MIMIs, we used another Gaussian LMM (Model 4) to test the effect of MIMIs in a given year (predictor) on the time dyads spent in close proximity in the consecutive year (response). Current proximity time, current CSI values, and the ID of the social group were included as fixed factors. Male identities and dyads were included as random factors. For this analysis we calculated the time male dyads spent in close proximity (≤1.5m). Here the proximity time following MIMI was not considered. To clarify whether MIMIs promote bond formation or whether only stronger bonded dyads are able to engage in MIMIs we ran a reversed model (Model 5) testing whether the strength of a dyadic social affiliative relationship (predictor) predicts the MIMI rate of the consecutive year (response). Social group, current MIMIs, and the future CSI were include as fixed factors. Male identities and dyad were included as random factors.

All models were run in R (version 3.2.2, R Core Team, 2015) using the function ‘lmer’ of the R package ‘lm4’ [85]. We derived the *P* value for each predictor in all three models by likelihood ratio tests using the R function ‘drop1’ [86]. To check that the assumptions of the models are fulfilled, we inspected visually the distribution of the residuals plotted against the fitted values [87]. For all three models the residuals were homogeneously distributed. Furthermore, we calculated for each predictor the variance inflation factor by using the function “vif” of the R package car [88]. VIFs in all our models were below 5 indicating that the collinearity between the predictors was not an issue [89]. Finally, we checked for model stability by excluding data points one by one from the data and by comparing the estimates derived with those obtained for the full model to check for model stability. All models were stable.

## Results

The number of male dyads in each group varied across the years between 21 and 55 (*mean*±SE = 39.8±5.4) due to migration and death. Across the observation periods we recorded in total 919 MIMIs. Across the study period all males were observed to engage in MIMIs. On average±SE 92.8±3.2% of all males engaged in a MIMI each observation period. The majority of MIMIs (79.3%) occurred in the first 10sec after two males approached (see S1 Table).

### MIMIs and time spent in close proximity

Across all observation periods, 97.1% of all male-male dyads approached each other at least once. In total, we recorded 16,550 approaches with an average±SE rate of 0.22±0.01 approaches per hour per dyad. 12.4% of all approaches were followed by a social interaction between the males. Out of the total number of approaches, 2.4% were followed by an aggression and 10.0% by an affiliative social interaction. More detailed information on the occurrence of behaviours upon approaches divided by whether dominant or subdominant males approached is given in S2 Table.

**Fig 1:**
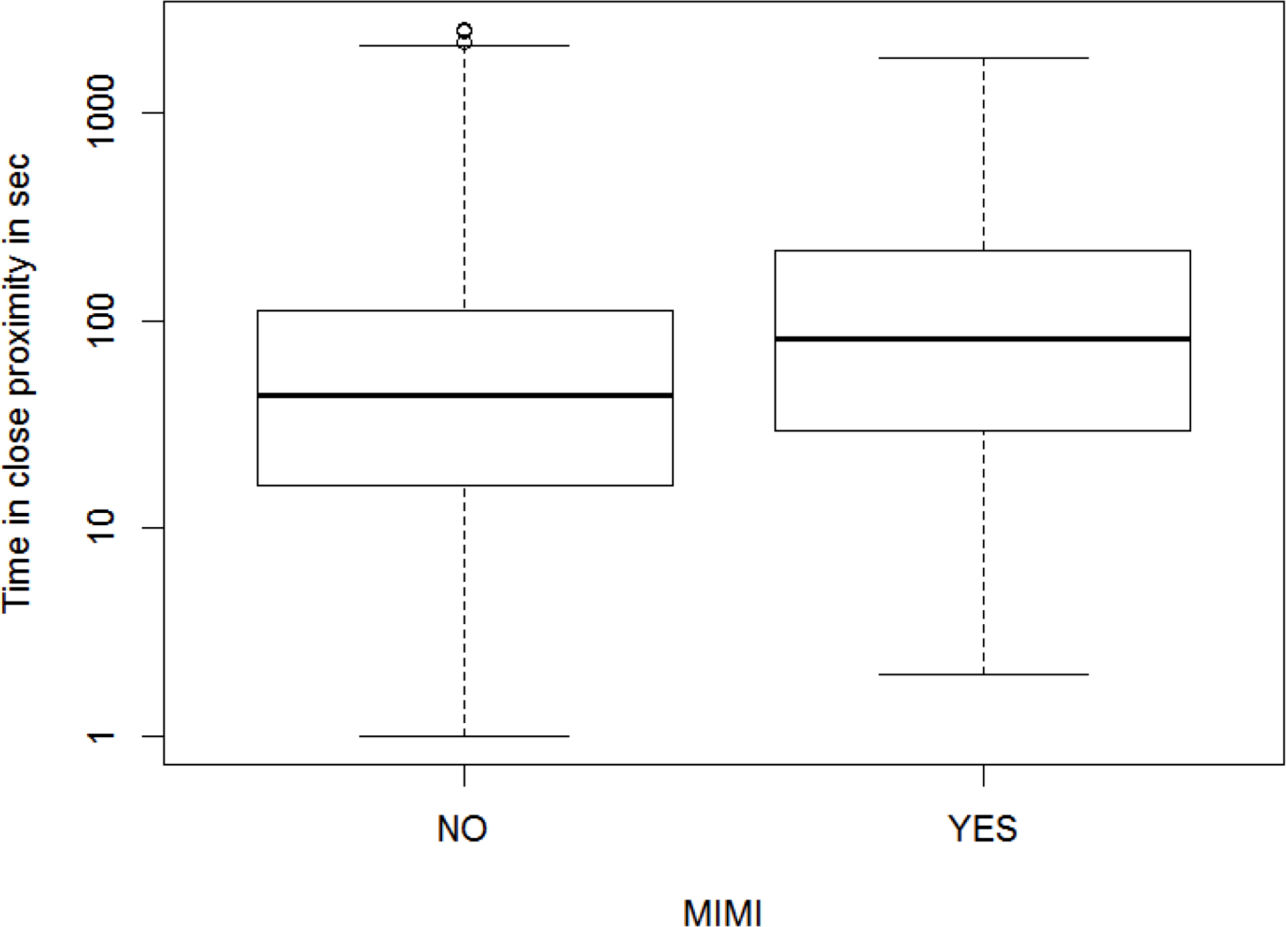
The effect of male-infant-male interactions (MIMIs) on time two males spent in close proximity after an approach by the subordinate in the dyad (N_Dyads_ = 186).

To test the impact of MIMIs on the subsequent time spent in close proximity right after the approach, we extracted all approaches where subordinate males approached, which resulted in a subset of 7,015 approaches. Here 13.5% of the approaches were followed by a social interaction; only 2.4% of the approaches were followed by an aggression whereas in 11.1% males engaged in an affiliative interaction (4.2% MIMIs). The interaction between CSI and MIMI did not significantly affect time spent in proximity (Model 1, N = 7015, Chi^2^ = 0.25, P = 0.618). Therefore we reran the model without this interaction. The new model was significantly different from the null model (Chi^2^ = 46.29, P < 0.001, R^2^ = 0.60) and showed that subordinate males who approached a male higher in rank spent significantly more time in close proximity if an approach was followed by a MIMI (average±SE = 186±15.94sec, range: 2–1,843sec) than after an approach without a MIMI (average±SE = 120±2.78sec, range: 0–2,524sec, Table 1, Fig 1). We found the same result if subordinate males got approached by more dominant males (Model 2, see S3 Table).

**Table 1:** Estimates±SE, Z and P values for the LMM (Model 1) ran to test whether male-infant-male interactions (MIMIs) have an effect on subsequent time spent in proximity after a subordinate male approached a dominant male.

Predictors	Estimates±SE	t	P
Intercept	-3.98±0.19	-21.44	<0.001
MIMI after approach	0.18±0.03	6.82	<0.001
CSI	-0.00±0–01	-0.7	0.497
Rank Distance	0.00±0.00	0.46	0.687
Social group	0.53±0.05	9.67	<0.001

Number of Observations = 7015; Number of Dyads = 186

The boxes indicate medians (thick line) and first and third quartiles. The whiskers indicate the 90th and 10th percentiles. The y-axis is plotted on a logarithmic scale.

### Effect of relationship strength on MIMI frequency

**Fig 2:**
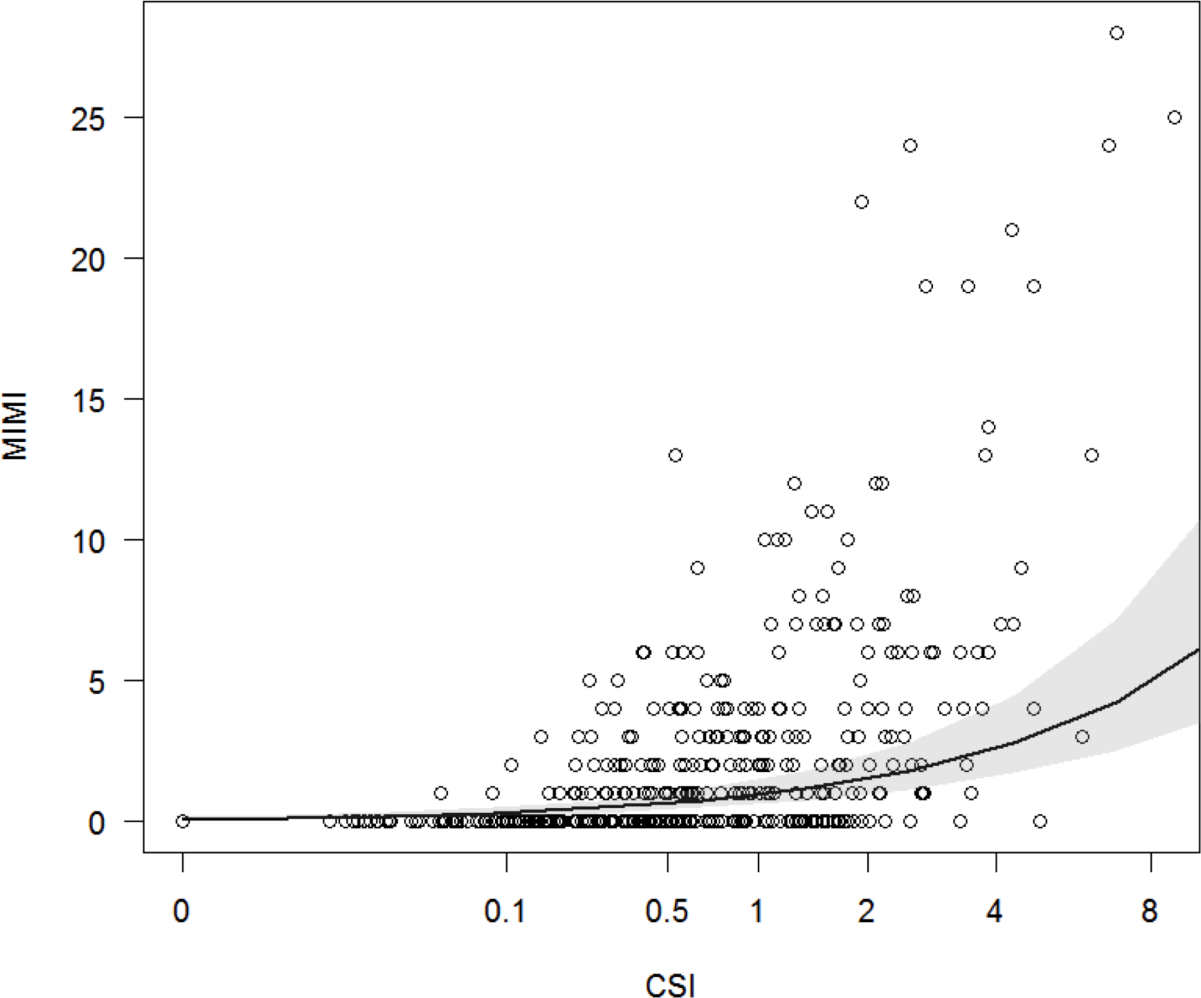
The occurrence of male-infant-male interactions (MIMIs) as a function of relationship strength (CSI). The CSI and the frequency of MIMIs were calculated for each dyad in each observation period. The black line depicts the relationship between CSI and MIMI as predicted by Model 3. The circles represent the raw data of 407 dyads and the grey area the 95% confidence interval of the model. The x-axis is plotted on a double square root scale (^0.25).

Model 3 was significantly different from the null model (Chi^2^ = 85.89, P <0.0001, R^2^ = 0.40). Dyadic relationship strength positively influenced the occurrence of MIMIs (N = 407 dyads, Model 3, Table 2, Fig 2). The stronger a relationship was, the more often males engaged in MIMIs. Each dyad handled an infant on average±SE 0.01±0.00 times per hour (range: 0–0.24, N = 407). Weakly affiliated dyads (CSI<1) engaged in MIMIs on average±SE 0.01±0.00 times (range: 0–0.09, N = 269) and strongly affiliated dyads with a CSI≥1 0.03±0.00 times (range: 0–0.24, N = 138), i.e. roughly once every 3 days. On average±SE each male had 4.57±0.26 (range: 0–9) different MIMI partners which indicates that at least some males were highly selective in their partner choice for MIMIs, as has been observed with other affiliative behaviours among these males [33].

**Table 2:** Estimates±SE, Z and P values for the GLMM (Model 3) run to test whether the strength of the affiliative relationship between two males (CSI) has an effect on how often they engage in male-infant-male interactions (MIMIs).

Predictors	Estimates±SE	z	P
Intercept	-4.28±0.43	-9.99	<0.0001
CSI	2.73±0.30	9.15	<0.0001

Number of dyads = 407 across periods. (A dyad can occur in several periods.)

### Long-term effect of MIMI on future time spent in close proximity

**Fig 3:**
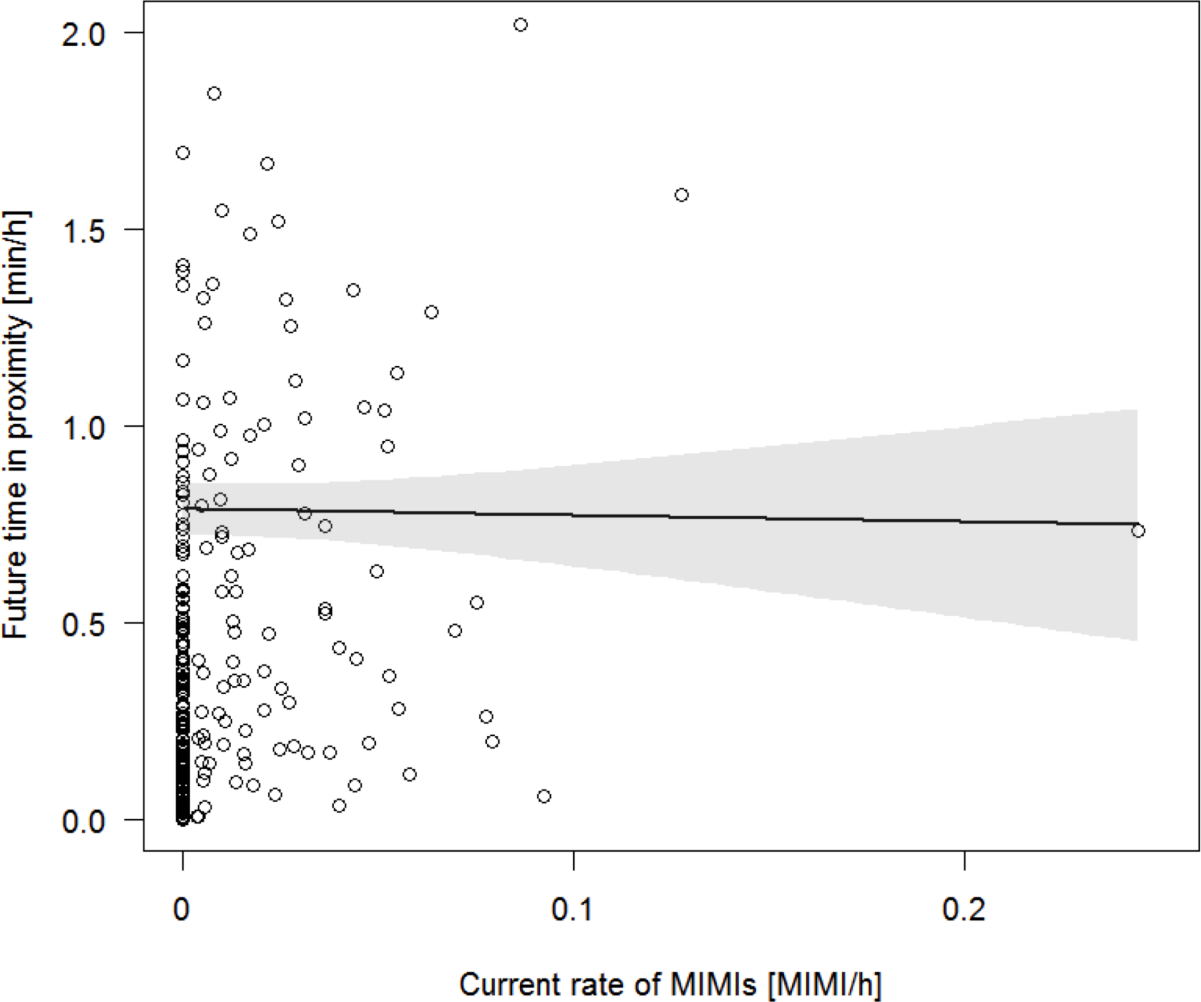
The effect of current male-infant-male interactions (MIMIs) on future proximity time. The black line depicts the relationship between current MIMI and future time in proximity as predicted by Model 4. The circles represent the raw data of 129 dyads and the grey area the 95% confidence interval of the model.

We found no evidence that the current rate of MIMIs predicted future proximity of a dyad (N = 240, Model 4). Model 4 was not significantly different from the null model (Chi^2^ = 0.234, P = 0.879, R^2^ = 0.36). This subset of our data contained a dyad which appeared as an outlier in that their rate of MIMI was almost twice as high as the rate of any other dyad (see Fig 3). Rerunning Model 4 after removal of the outlier did not change the results.

We found no evidence that the strength of a dyadic affiliative relationship predicted the MIMI rate of the consecutive year (N = 240, Model 5). The model was not significantly different from the null model (Chi^2^ = 0.186, P = 0.666, R^2^ = 0.48).

## Discussion

The results of our correlational analysis suggest that wild male Assamese macaques may use male-infant-male interactions (MIMIs) to form and in particular to maintain their affiliative relationships rather than solely as an agonistic buffering mechanism. The closer two males were affiliated the more often they engaged in MIMIs suggesting that like allo-grooming mutual infant handling might serve to maintain social bonds. Handling an infant upon approach with dominant males allowed subordinate males to subsequently spend more time in close proximity to higher ranking males and to possibly increase the likelihood to engage in other affiliative interactions and thereby strengthening their affiliative relationship.

Our results that MIMIs increase the time male-male dyads spend in close proximity add to the findings of other macaque studies [64, 66, 90, 91]. Subordinate male Tibetan macaques are more likely to approach males higher in rank if they engaged in MIMIs [64]. Although most MIMIs in Tibetan macaques occurred in an affiliative rather than aggressive context, the increase in spatial tolerance is interpreted as reflecting aggression avoidance within the group. Since the MIMI was often followed by grooming [64] the increased time males spent in close proximity after MIMIs, might increase the likelihood to engage in other affiliative interactions, which in turn might shape affiliative relationships between males paralleling results of our study. Male Assamese macaques stayed on average up to 50% longer in close proximity to higher ranking males, if they had engaged in a MIMI upon their approach. Interestingly, this was irrespective of their affiliative relationship strength indicating that every subordinate male had the same beneficial outcome of MIMIs regardless of the relationship they shared with the higher ranking male.

As in other species (e.g. female baboons [29]; male chimpanzees [6, 22]), strongly affiliated male Assamese macaques spend significantly longer time in close proximity than weakly affiliated dyads [33] and therefore should not need to engage in a MIMI simply to increase their time in close proximity with each other. Interestingly, male Assamese macaques sharing a stronger affiliative relationship (CSI≥1) were about five times more often involved in MIMIs than male-male dyads with a weaker relationship (CSI<1) and relationship strength was positively associated with MIMI frequency even after controlling for the time they spent in 5m spatial proximity. These results, together with the finding that the dyadic frequency of MIMIs influences future cooperation in Barbary macaque males [74] and that affiliative male-infant relationships are initiated by infants rather than by the males of this study [92], support the hypothesis that MIMIs function to form and reinforce male social bonds [65]. In this sense, MIMIs in macaque males may functionally parallel greetings exchanged between baboon males that are also proposed as a bonding mechanism enhancing a male’s willingness to cooperate [65, 93, 94].

Our results do not provide direct support for the idea that MIMIs function also in bond formation. We found no evidence that current MIMIs predicted the time two males spend in close proximity in the future, which may have been caused by the long-term stability of male relationships in our dataset. Throughout this study period we did not observe adult males immigrating into our study groups. Thus the relationships among males may have had established before the onset of the study. Therefore, we cannot rule out a role of MIMIs in bond formation, especially since we found an effect of MIMIs on immediate proximity time even for weakly affiliated partners which would be the pre-condition for establishing new bonds. To rule out circularity, i.e. whether MIMIs promote social bonds or whether social bonds promote the engagement in MIMIs, we found no evidence that bond strength of a male dyad influences the MIMI rate in the following year.

Having closely bonded male partners is an important factor enhancing male Assamese macaques’ fitness. Males of this species form coalitions with closely bonded partners to attain and maintain high social status which in turn regulates their paternity success [7, 95]. Yet the opportunity to bond might be limited in Assamese macaques since a male approaches any other male on average only 0.22 times per hour and only 10% of these approaches are followed by an affiliative social interaction. A male’s time budget and its ability to devote time to establish and maintain affiliative relationships with other males is restricted by time devoted to other activities such as bonding with females [19, 32, 96] and infants [92, 97–99], sexual consortships [100–104] and foraging [105–107]. In light of these time constraints, males should optimize the little time they can afford to invest in bonding. In this respect, MIMIs might be more efficient than grooming. Grooming is an important affiliative bonding behaviour in primates (reviewed in [108]) and has been often used as the main measure of the strength and the quality of dyadic relationships (e.g. [22, 29, 33, 109]). Yet, quantitative data suggest that by the same standards MIMIs might be as important as grooming in the bonding of male Assamese macaques since a similar percentage of approaches are followed by grooming (2.9%) or MIMIs (3.7%). Grooming is time consuming and is a directional behaviour. In contrast, MIMIs are brief contact behaviours with no specific directionality and might, therefore, serve as an appropriate additional behaviour to maintain affiliative relationships by alleviating the constraints related to reciprocity needs. Conclusive tests of the bond maintenance function both of grooming and MIMIs would require stopping dyads from performing these behaviours and these behaviours only and tracking the change in their relationship quality on other scales.

Similar to grooming, the effect of MIMIs on bonding may be mediated by underlying neurochemical mediators like endorphins or oxytocin [77, 110,111]. The hormonal release of oxytocin alleviates stress but also increases prosocial behaviour and enhances trust between individuals, thereby enhancing the bonding process [112, 113]. Similarly, affiliative physical contact such as grooming leads to an endorphin release followed by an activation of the neural reward system in association with a feeling of pleasure [110, 114]. Due to rapid degradation of endorphins individuals are motivated to continue engaging in social contact leading to the maintenance and reinforcement of affiliative relationships [110]. Such a possible increase of partner-specific positive emotions might constitute a bookkeeping system which triggers future affiliative (e.g. MIMI, grooming) and cooperative interactions (e.g. coalitions) with specific partners [115–118], thereby maintaining and strengthening affiliative relationships over time. It remains to be shown whether males exhibit more strongly increased oxytocin or endorphin levels after contact involving MIMIs than after other friendly body contact without an infant being involved.

To conclude, MIMIs so far were mainly linked to the agonistic buffering hypothesis stating that MIMIs enable subordinate males to approach males higher in rank and reducing the likelihood of subordinates to receive aggression from a higher ranking male when in close proximity [56, 69, 70–72]. Paul et al. [65] suggested the possible influence of MIMIs in male-male social bonding. In accordance with this notion, the results of our study show that MIMIs might be an important behavioural mechanism in male Assamese macaques that functions on a proximate level with an increase in proximity time and possibly a change in attitude as well as on an ultimate level to establish and maintain social bonds and thereby enhance immediate and future benefits.

## Supporting information

S1 Table Latency of MIMI occurrence after an approach. *Total No. of MIMIs N = 614.
(DOCX)

S2 Table Occurrence of behaviours upon approach. Out of the total number of approaches (N = 16650), 1422 approaches between two males occurred simultaneously.
(DOCX)

S3 Table MIMIs and time spent in close proximity after an approach of a dominant male. Estimates±SE, Z and P values for the LMM ran to test whether MIMIs have an effect on time spent in proximity after a dominant male approached a lower ranking male.
(DOCX)
