# Supplementary material for: Triadic male-infant-male interaction serves in bond maintenance in male Assamese macaques
Source: PLoS One. 2017 Oct 18;12(10):e0183981. doi: 10.1371/journal.pone.0183981 (PMC5646793; doi:10.1371/journal.pone.0183981)
Supplement: S1 Table — *Total No. of MIMIs N = 614. (DOCX) [file pone.0183981.s001.docx]

## S1 Table, Latency of MIMI occurrence after an approach

| MIMIs | No. of MIMIs*, in (%) |
| --- | --- |
| 0-10sec after approach | 478 (79.32) |
| 11–30sec after approach | 52 (8.47) |
| 31–60sec after approach | 26 (4.24) |
| 61–300sec after approach | 43 (7.00) |
| > 300sec after approach | 6 (0.98) |

*Total No. of MIMIs N = 614
