# Supplementary material for: Triadic male-infant-male interaction serves in bond maintenance in male Assamese macaques
Source: PLoS One. 2017 Oct 18;12(10):e0183981. doi: 10.1371/journal.pone.0183981 (PMC5646793; doi:10.1371/journal.pone.0183981)
Supplement: S2 Table — Out of the total number of approaches (N = 16650), 1422 approaches between two males occurred simultaneously. (DOCX) [file pone.0183981.s002.docx]

## S2 Table, Occurrence of behaviours upon approach

|  | | | Total No.of approaches and interactions | Subordinate approaches dominant | Dominant approaches subordinate |
| --- | --- | --- | --- | --- | --- |
| Total No. of approaches |  | | 16650 (100%) | 7015 (100%) | 8213 (100%) |
| No interaction |  | | 14479 (86.96%) | 6071 (86.54%) | 7324 (89.18%) |
| Social interaction | Affiliation | Embrace | 168 (1.01%) | 77(1.10%) | 70 (0.85%) |
|  |  | Mount | 402 (2.42%) | 172 (2.45%) | 188 (2.89%) |
|  |  | Groom | 481 (2.88%) | 235 (3.35%) | 190 (2.31%) |
|  |  | MIMI | 614 (3.69%) | 295 (4.21%) | 254 (3.09%) |
|  |  | **Total** | **1665 (10.00%)** | **779 (11.11%)** | **703 (9.58%)** |
|  | Aggression | Bite | 25 (0.15%) | 12 (0.17%) | 12 (0.15%) |
|  |  | Chase | 89 (0.53% | 27 (0.39%) | 44 (0.54%) |
|  |  | Lunge | 151(0.91% | 60 (0.86%) | 71 (0.86%) |
|  |  | Push & pull | 92 (0.55%) | 42 (0.60%) | 23 (0.28%) |
|  |  | Slap | 49 (0.29%) | 24 (0.34%) | 46 (0.56%) |
|  |  | **Total** | **406 (2.44%)** | **165 (2.35%)** | **196 (2.39%)** |

Out of the total number of approaches (N = 16650), 1422 approaches between two males occurred simultaneously.
