# Supplementary material for: Triadic male-infant-male interaction serves in bond maintenance in male Assamese macaques
Source: PLoS One. 2017 Oct 18;12(10):e0183981. doi: 10.1371/journal.pone.0183981 (PMC5646793; doi:10.1371/journal.pone.0183981)
Supplement: S3 Table — Estimates±SE, Z and P values for the LMM ran to test whether MIMIs have an effect on time spent in proximity after a dominant male approached a lower ranking male. (DOCX) [file pone.0183981.s003.docx]

## S3 Table, MIMIs and time spent in close proximity after an approach of a dominant male

The interaction between CSI and MIMI did not significantly affect time spent in proximity (Model 2, N=8213, Chi²=0.20, P=0.65). Therefore we reran the model without this interaction. The model was significantly different from the null model (Chi²=36.94, P < 0.001, R^2^=0.63). MIMIs had a positive effect on time spent in proximity after an approach.

Estimates±SE, Z and P values for the LMM ran to test whether MIMIs have an effect on time spent in proximity after a dominant male approached a lower ranking male.

| Predictors | Estimates±SE | t | P |
| --- | --- | --- | --- |
| Intercept | -3.96±0.19 | -20.80 | <0.001 |
| MIMI after approach | 0.17±0.03 | 6.09 | <0.001 |
| CSI | 0.00±0.01 | 0.62 | 0.54 |
| Rank Distance | -0.00±0.00 | -1.99 | 0.06 |
| Social group | 0.52±0.06 | 8.77 | <0.001 |

Number of Observations = 8213; Number of Dyads = 187
